# Effect of the Internal Humidity of Concrete on Frost Resistance and Air Void Structure under Different Low Temperature Conditions

**DOI:** 10.3390/ma15155225

**Published:** 2022-07-28

**Authors:** Xueliang Ge, Mingyong Ke, Weibao Liu, Heng Wang, Cairong Lu, Guoxing Mei, Hu Yang

**Affiliations:** 1 Nanjing Hydraulic Research Institute, Nanjing 210029, China; myke@nhri.cn (M.K.); wbliu@nhri.cn (W.L.); wangheng@nhri.cn (H.W.); crlu@nhri.cn (C.L.); gxmei@nhri.cn (G.M.); yangh@nhri.cn (H.Y.); 2 State Key Laboratory of Hydrology-Water Resources and Hydraulic Engineering, Nanjing 210029, China; 3 Research Center for Climate Change, Ministry of Water Resources of China, Nanjing 210029, China; 4 Research Center on New Materials in Hydraulic structures, Ministry of Water Resources of China, Nanjing 210024, China

**Keywords:** concrete, internal humidity, freeze temperature, frost resistance, air void structure

## Abstract

From the perspective of combining macroscopic and microscopic properties, this paper simulates the freeze–thaw cycle process at different freezing low temperatures based on the climate simulation equipment and by setting the curing conditions with different temperatures and relative humidity to produce different moisture conditions in concrete. The frost resistance properties and microscopic air void performance of concrete with different internal water content under different freezing low temperatures in freeze–thaw cycles were systematically studied. The results show that the higher the internal water content of concrete, the more obvious the mass loss rate and dynamic elastic modulus loss of concrete in the freeze–thaw process, and the more serious the deterioration of the air void parameter performance of the air-entraining agent introduced into concrete, which is manifested as the average bubble diameter and bubble spacing factor become larger and the bubble specific surface area decreases. In addition, in the case of the same internal moisture content of concrete, the freezing temperature used in the freeze–thaw cycle also has an important impact on the frost resistance of concrete and air void parameters; the lower the freezing temperature used, the more significant the decline in the frost resistance of concrete, the more obvious the deterioration of air void parameters.

## 1. Introduction

In order of importance, the factors causing the damage to concrete structures are reinforcement corrosion, freeze–thaw and seawater erosion. Freeze–thaw is one of the most representative factors affecting the durability of concrete [1]. According to the first Chinese water census bulletin in 2013 [2], more than 98,000 various types of reservoirs have been built in China, with a total capacity of about 932,312 million m^3^. These reservoirs are distributed in different climatic regions of China, and the number of freeze–thaw cycles and freeze–thaw temperature conditions they undergo each year are different. The summary report of the survey on the durability of concrete by the former Ministry of Water and Power of China in 1985 stated: freeze–thaw damage of hydraulic concrete was present in almost 100% of the projects in the three northern regions (i.e., northeast, north and northwest China). For cold regions, freeze–thaw action is the main factor leading to structural performance damage of hydraulic concrete, and cold regions of hydraulic buildings suffer from low-temperature frost damage environment is much lower than the current standard test method for concrete frost resistance −17 °C conditions used. Freeze–thaw action has become one of the most representative factors affecting the deterioration of concrete durability of hydraulic buildings in the cold regions of China.

Existing research [3] shows that there is a limited water saturation for freeze–thaw damage of almost all porous materials, and concrete as a porous material also has the problem of limited water saturation. The freeze–thaw critical water value method proposed by Fagerlund [4] argues that the degree of freeze–thaw damage of concrete is closely related to the internal water content of concrete, and there is a critical value for the free water content of concrete, and the degree of saturation is not reached, even in the cold environment, concrete will not receive freeze damage, and when the water content reaches the critical value, concrete will be rapidly damaged. According to the hydrostatic pressure theory of T.C. Powers [5], the volume expansion of capillary pore water causes freezing damage at low temperatures, while the freezing temperature of pore water depends on the pore size, and the smaller the pore size, the lower the freezing temperature [6]. Since T.C. Powers [7] established the theory of concrete pore structure and frost resistance, researchers have conducted a large number of studies on the relationship between bubble characteristic parameters and concrete frost durability based on this theory [8,9,10,11,12,13], but these studies are still inconclusive and the research conclusions are very different. There is no conclusion on the reasonable bubble spacing for improving the frost resistance of concrete, etc. [14], and among the available research results [15], the measured bubble spacing in hardened concrete varies widely, ranging from 200 μm to 800 μm. In addition, in experimental freeze–thaw studies of concrete materials, researchers have more often considered freeze–thaw damage of concrete under fixed temperature cycles, temperature drop rates and temperature drop amplitudes [16], and the frost resistance has focused mainly on macroscopic properties, and few studies have focused on the frost resistance of concrete under different temperature drop amplitudes and temperature drop rates [17,18,19].

Therefore, in this study, three low-temperature conditions of −17 °C, −30 °C and −40 °C were simulated using climate simulation equipment for concrete freeze–thaw tests, so that the central temperature of the concrete specimens reached −17 °C, −30 °C and −40 °C during the freeze-cooling process and 8 °C during the warming process of concrete thawing. The influence of the internal water content of concrete on the mass loss and dynamic elastic modulus change during the freeze–thaw process of concrete was carried out under these freeze–thaw conditions. At the same time, the changes of microscopic bubble structure parameters, including the average diameter of bubbles, bubble spacing coefficient and bubble specific surface area, were investigated in concrete with different internal moisture conditions during freeze–thawing under different low temperature freeze–thaw conditions.

## 2. Materials and Methods

### 2.1. Raw Materials

The raw materials used in this study include P·O 42.5 Portland cement conforming to the requirements of Chinese standard *Common Portland cement GB 175-2007*, Class F fly ash conforming to the requirements of Chinese standard *Fly ash used for cement and concrete GB/T 1596-2017*, a naphthalene water reducing agent and Air-202 air-entraining agent (solids content of 2%) conforming to the requirements of Chinese standard *Concrete admixtures GB 8076-2008*. Aggregate includes natural sand, small stones with particle size of 5–20 mm and medium stones with particle size of 20–40 mm. The natural sand had a fineness modulus of 2.71 and surface dry water absorption of 1.26%. The surface dry water absorption of small and medium stones was 1.0% and 0.8%, respectively.

### 2.2. Concrete Mix Proportion

The concrete mix is shown in Table 1. Concrete specimens were formed and maintained according to the Chinese water resources industry standard *Test code for hydraulic concrete SL/T 352-2020*. The slump of the concrete mix was controlled by 70 ± 20 mm, the air content was controlled by 5.5 ± 0.5%, and the 28 d strength grade of the concrete specimens was C30, and the frost resistance design grade was F300.

### 2.3. Methods

The size of concrete specimens used in the test was 100 mm × 100 mm × 400 mm. Firstly, 63 concrete specimens were standard cured at 20 °C temperature and 90% relative humidity for 28 days. Secondly, at the end of standard curing, the 63 concrete specimens were equally divided into three Groups A, B and C. The specimens in Groups A, B and C were further cured according to the temperature, relative humidity and curing time in Table 2 to produce different internal water content conditions in the concrete specimens.

Three specimens were taken out from the three Groups of concrete A, B and C, weighed out the initial weight and calculated the average value m_0_. Then these concrete specimens weighed with the initial weight were baked to constant weight in the oven at 100 °C, cooled to room temperature, weighed again and the average value was calculated as m_1_. The moisture content of the concrete specimens from the three Groups A, B and C could be calculated quantitatively as (m_0_ − m_1_)/m_0_.

**Figure 1:**
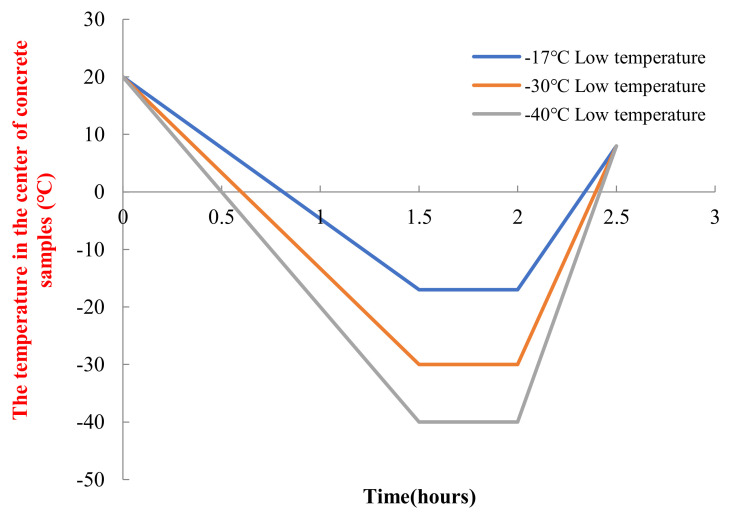
Temperature curve of a freeze–thaw cycle under different low temperature.

The other eighteen concrete specimens in the three Groups of A, B and C were subjected to freeze–thaw cycle test with reference to the rapid freeze–thaw method in the Chinese water industry standard *SL/T 352-2020*. The freeze–thaw test was carried out using the developed extreme climate simulation equipment. It can provide temperatures from −70 °C to 150 °C with a temperature control accuracy of 0.1 °C. It can also provide a relative humidity environment from 10% to 98%, with a relative humidity control accuracy of 1%. The freezing and thawing tests were conducted using concrete freezing low temperatures of −17 °C, −30 °C and −40 °C. The temperature rise and temperature drop curves of the freezing and thawing tests are shown in Figure 1.

The mass and dynamic elastic modulus of the concrete was tested after every fifty freeze–thaw cycles. The mass of concrete was tested using an electronic scale with a maximum weighing of 10 kg and induction of 5 g. Dynamic modulus of elasticity tests were performed using dynamic modulus of elasticity testing equipment with frequencies ranging from 100 Hz to 10,000 Hz. The mass and dynamic elastic modulus of concrete specimens was tested in accordance with the Chinese water resources industry standard *SL/T 352-2020*. The dynamic elastic modulus characterizes the propagation properties of elastic waves in concrete and can be used to evaluate the internal damage of concrete caused by freeze–thaw. The dynamic elastic modulus can be calculated according to the following equation:

(1) $$P_{n}=\frac{f_{n}^{2}}{f_{0}^{2}}\times 100$$

where $P_{n}$ is the relative dynamic elastic modulus after n freeze–thaw cycles, %. $f_{0}^{2}$ is the natural vibration frequency of concrete specimen before freeze–thaw test, Hz. $f_{n}^{2}$ is the natural frequency of concrete specimen after n freeze–thaw cycles, Hz.

At the end of every 100 freeze–thaw cycles, concrete specimens were sectioned for micro air void parameter test. The slice sample for air void parameters test was made according to the relevant requirements of the *ASTM standard C457*. The size of air void parameter test slice is 100 mm × 100 mm × 20 mm. In order to enhance the contrast of slices and improve the test accuracy, a contrast enhancement step to make air voids appear white and aggregates and paste appear black was conducted. Firstly, paint the sliced surface black with a black marker pen, in the process of coloring, be careful not the fill voids with ink. Then sprinkle about one t-spoon of white fine-grained BaSO_4_ on the black surface. Take a small, very hard rubber stopper and tap the white powder into the air voids. Tap for about 2 min until all voids appear filled. In the final step voids or cavities in aggregate as well as obvious cracks are colored black under the stereomicroscope using a marker pen. If very large voids are found in the aggregate they may be covered by a piece of black tape. The testing of microscopic air void parameters was performed by Rapidair 3000 system. The test results are automatically given by the software of Rapidair 3000 after computer processing. The air void parameters were tested according to ASTM C457/C457M-16 *Microscopical Determination of Parameters of the Air-Void System in Hardened Concrete*.

## 3. Results and Discussion

### 3.1. Concrete Properties and Internal Moisture Content

The 28 days compressive strength of the concrete prepared according to the proportions in Table 1 was 36.2 MPa, with a frost resistance grade of F300. The water content of the concrete specimens in Groups A, B, and C before the freeze–thaw cycle test was 15%, 8%, and 4% on average, respectively. Therefore, further curing of the concrete with different temperature and relative humidity environmental conditions after completing the 28 d standard curing can change the water content conditions inside the concrete for the purpose of the test. The water content of the concrete specimens in Groups A, B and C decreased with the increase in the curing temperature and the decrease in the relative humidity.

### 3.2. Mass Loss and Dynamic Elastic Modulus Change

**Figure 2:**
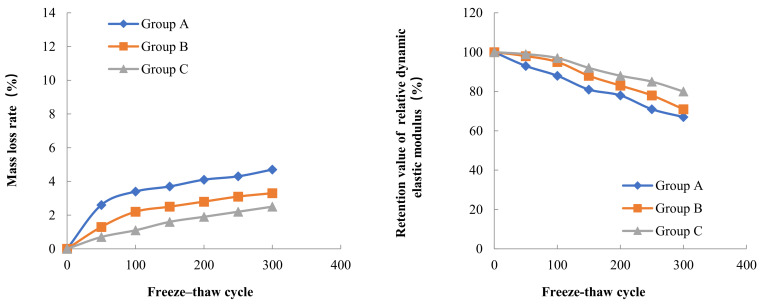
Frost resistance of concrete with different internal humidity (−17 °C freezing temperature).

**Figure 3:**
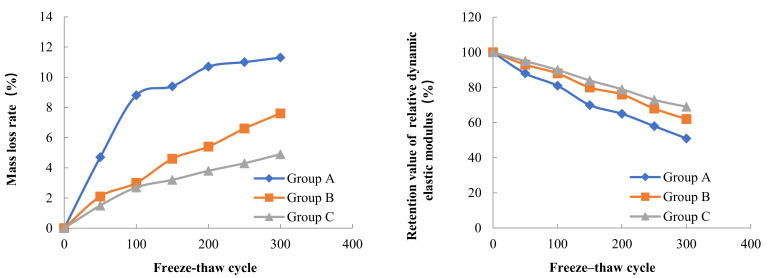
Frost resistance of concrete with different internal humidity (−30 °C freezing temperature).

**Figure 4:**
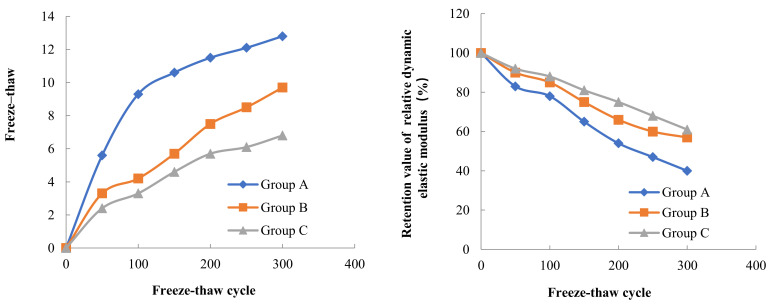
Frost resistance of concrete with different internal humidity (−40 °C freezing temperature).

Three concrete specimens were taken from each Group for testing of mass loss and dynamic elastic modulus loss. The initial mass and the initial dynamic elastic modulus of the concrete specimens were tested before the freeze–thaw cycle experiment. The mass and dynamic elastic modulus of each concrete specimen was tested again after every 50 freeze–thaw cycles. The results of the mass loss rate and dynamic elastic modulus loss of concrete in three Groups A, B and C during different low temperature freeze–thaw cycles are shown in Figure 2, Figure 3 and Figure 4.

**Figure 5:**
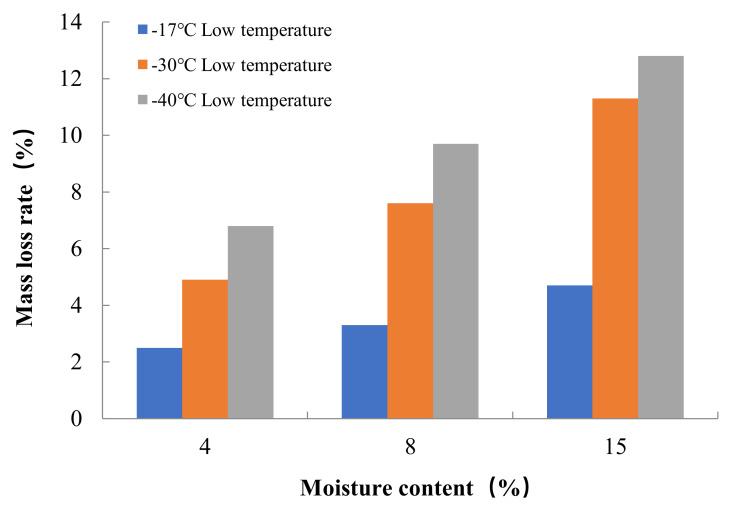
Relationship between internal moisture content and mass loss rate of concrete for freeze–thaw cycle test.

The results of freeze–thaw cycle experiments showed that the mass loss of concrete decreased with the increase in water content. After three hundred freeze–thaw cycles at a freezing temperature of −17 °C, the mass loss rates of concrete in Groups A, B and C were 4.7%, 3.3% and 2.5%, respectively. According to Powers’ hydrostatic pressure theory, the concrete with high internal water content can contain more capillary water freezing under the same freezing low temperature condition, forming a larger hydrostatic pressure in the pore, which leads to more serious freeze–thaw damage in the concrete with high internal water content. The test also found that the freezing low temperature had an important effect on the mass loss of concrete in the freeze–thaw cycle, when the freezing temperature decreased from −17 °C to −30 °C, the mass loss rate of concrete in Groups A, B and C increased by 140%, 130% and 96%, respectively, on the basis of the freezing mass loss rate at −17 °C. When the freezing temperature was further reduced from −30 °C to −40 °C, the mass loss rate of concrete in Groups A, B, and C increased by 39%, 28%, and 13% on the basis of the freezing mass loss rate at −30 °C. As the freezing temperature decreases, the cooling rate gradually accelerates, the water pressure inside the concrete pore will increase, when the water pressure exceeds the tensile strength of concrete, the concrete internal pore wall rupture occurs, therefore, the lower the freezing temperature, the more serious freeze–thaw damage occurs in concrete. The relationship between the internal water content of concrete and the mass loss rate after 300 freeze–thaw cycles at different low temperatures is shown in Figure 5.

**Figure 6:**
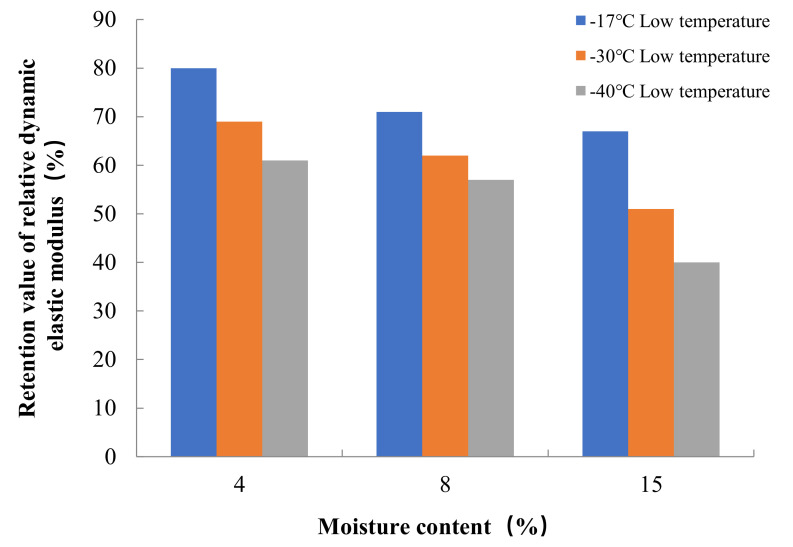
Relationship between internal moisture content and relative dynamic elasticity modulus retention value of concrete for freeze–thaw cycle test.

The relative dynamic elasticity modulus retention value of concrete decreases with the increase in water content inside the concrete. At the freezing temperature of −17 °C, the relative dynamic elasticity modulus retention values of concrete in Groups A, B, and C were 40%, 57%, and 61% after three hundred freeze–thaw cycles, respectively. Similarly, the relative dynamic elasticity modulus retention values of concrete also decreased with the decrease in freezing temperature. When the freezing temperature decreased from −17 °C to −40 °C, the relative dynamic elasticity modulus retention values of concrete in Groups A, B and C after three hundred freeze–thaw cycles decreased by 40%, 20% and 24%, respectively, on the basis of the relative dynamic elasticity modulus retention values at −17 °C freezing temperature. The dynamic elastic modulus mainly characterizes the propagation of elastic waves inside the concrete, and this propagation is directly related to the damage inside the concrete. From the analysis of the mass loss and the internal water content of the concrete as well as the freezing low temperature, it is clear that the greater the internal water content and the lower freezing temperature, the more serious the freezing damage of the concrete is, and thus the lower the relative dynamic elasticity modulus retention value of the concrete. The relationship between the internal water content of concrete and the relative dynamic elasticity modulus retention value after 300 freeze–thaw cycles at different low temperatures is shown in Figure 6.

### 3.3. Air Void Parameters

**Figure 7:**
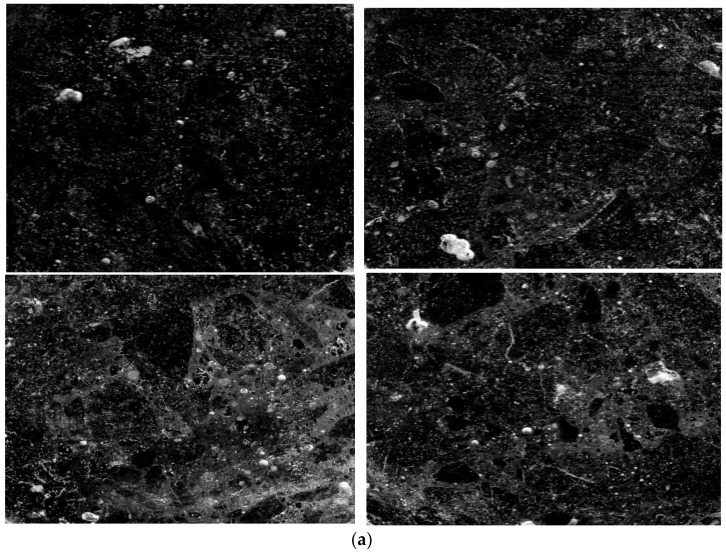

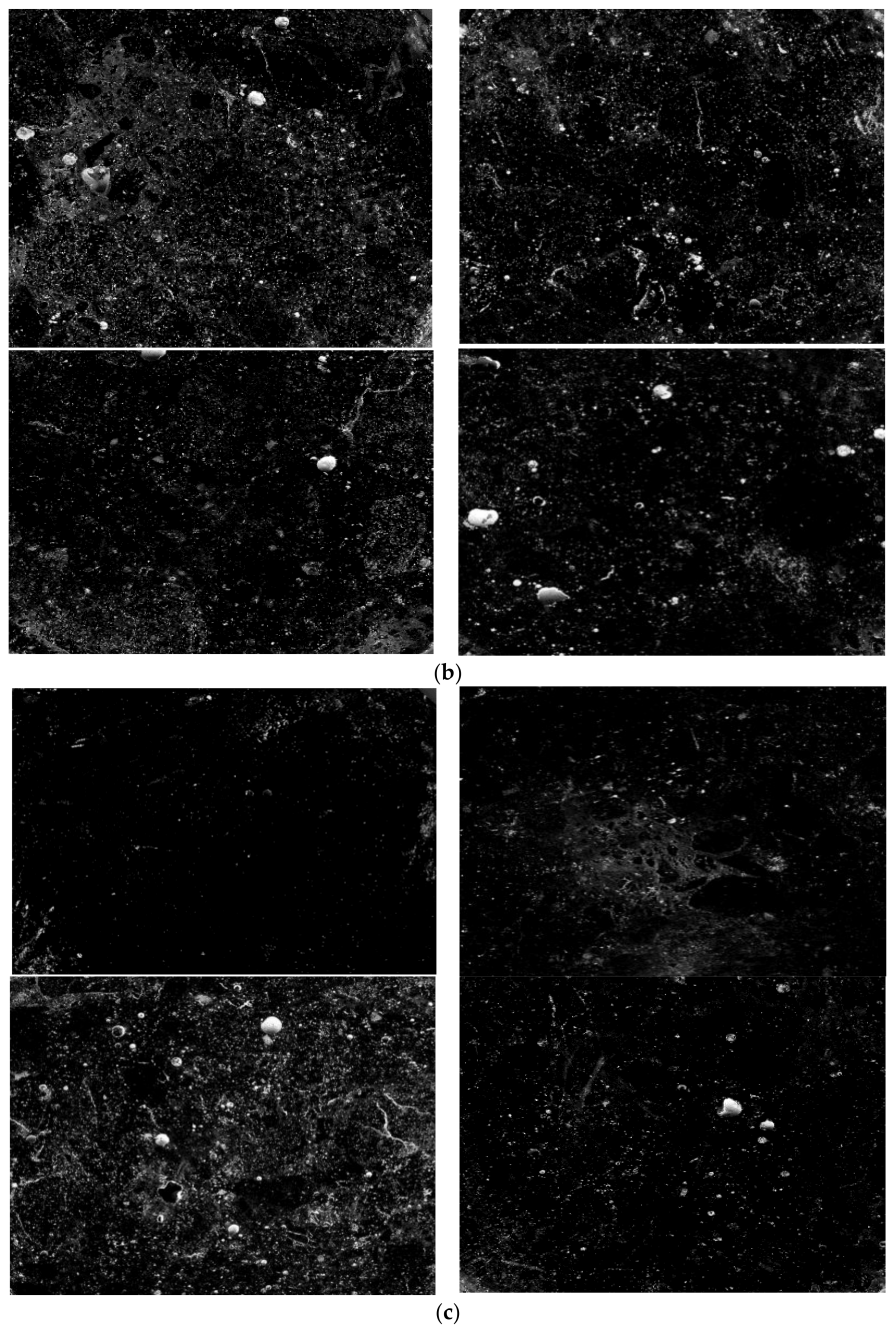
The prepared concrete cut plane samples for air void parameters test. (**a**) 15% moisture content concrete cut plane sample (Group A); (**b**) 8% moisture content concrete cut plane sample (Group B); (**c**) 4% moisture content concrete cut plane sample (Group C).

Air-entraining agents can introduce a large number of tiny bubbles in concrete to improve the frost resistance of concrete [20,21]. The characteristics of the introduced bubbles can be described by air void parameters, which mainly include the average bubble diameter, bubble specific surface area, and bubble spacing coefficient. The air void parameters have an important influence on the frost resistance of concrete [22,23,24,25]. After every hundred freeze–thaw cycles, the air void parameters of three Groups A, B and C of concrete were tested. The concrete cut plane samples used for air void parameters testing are shown in Figure 7.

The initial values of the air void parameters of the concrete in Groups A, B and C were tested before the freeze–thaw test, and the air void parameters of the concrete in Groups A, B and C were tested again at the end of every 100, 200 and 300 freeze–thaw cycles during the different low temperatures freeze–thaw tests. The changes of the air void parameters of the concrete in Groups A, B and C are shown in Table 3.

According to the test data in Table 3, before the freeze–thaw test, there was no significant difference in the air void parameters of concrete with different internal moisture contents in Groups A, B and C. The average diameter of bubbles ranged from 153 μm to 156 µm, the spacing factor of bubbles ranged from 175 µm to 178 µm, and the specific surface area of bubbles was around 40 mm^2^/mm^3^.

**Figure 8:**
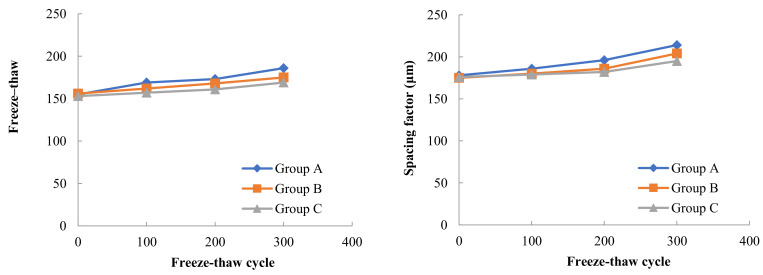

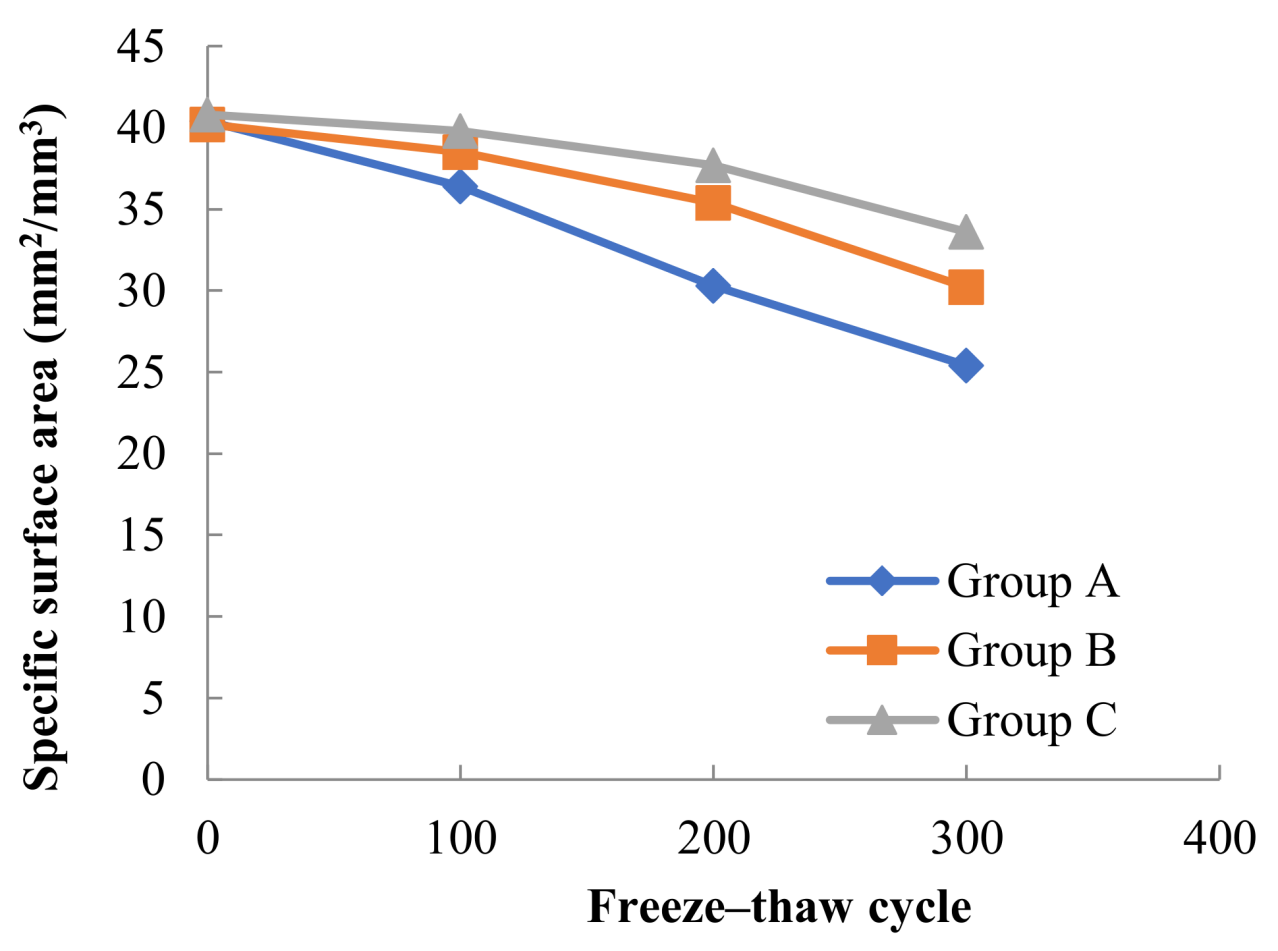
Variation of air void parameters for concrete with different internal water content (−17 °C freezing temperature).

The variation pattern of air void parameters with the number of freeze–thaw cycles for three Groups of concrete with different moisture contents in A, B and C under the freeze–thaw cycle test at −17 °C freezing low temperature and 8 °C melting temperature is shown in Figure 8. In the test, it was found that the internal water content of concrete had some influence on the internal bubble parameters of concrete during freeze–thaw cycles. Comparing the freeze–thaw tests of concrete in Groups A, B and C frozen at −17 °C and thawed at 8 °C, it can be seen that the general trend of bubble parameter changes was that the average diameter of bubbles increased, the spacing factor between bubbles increased and the specific surface area of bubbles decreased as the number of freeze–thaw cycles increased. With the increase in the internal water content of concrete, the average diameter of bubbles increased from 169 µm in Group C to 175 µm in Group B and then to 186 µm in Group A after 300 freeze–thaw cycles. The bubble spacing factor increased from 195 µm in Group C to 204 µm in Group B and then to 214 µm in Group A. The specific surface area of the bubbles decreased from 33.6 mm^2^/mm^3^ in Group C (4% water content of concrete) to 30.2 mm^2^/mm^3^ in Group B (8% water content of concrete) and then to 25.4 mm^2^/mm^3^ in Group A (15% water content of concrete).

**Figure 9:**
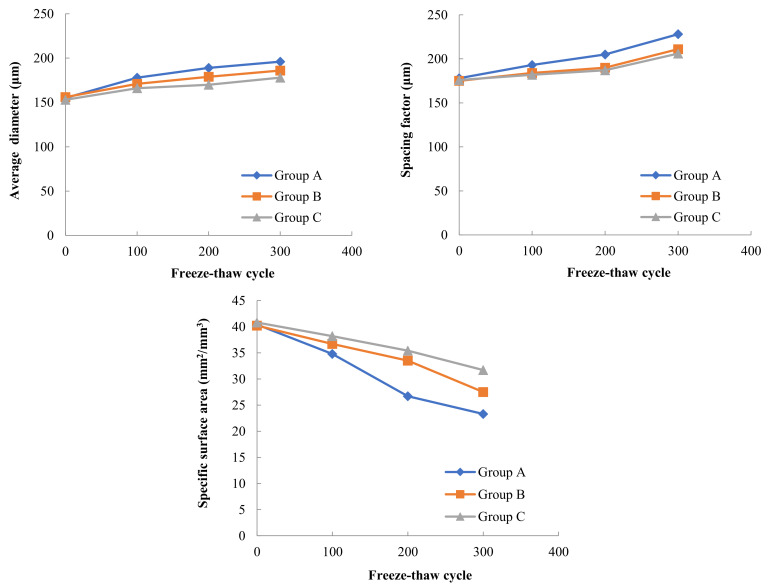
Variation of air void parameters for concrete with different internal water content (−30 °C freezing temperature).

The variation pattern of air void parameters with the number of freeze–thaw cycles for three Groups of concrete with different moisture contents in A, B and C under the freeze–thaw cycle test at −30 °C freezing low temperature and 8 °C melting temperature is shown in Figure 9.

In the freeze–thaw test with −30 °C freezing and 8 °C thawing, the changes of the average diameter of bubbles, spacing factor and bubble specific surface area in concrete with the number of freeze–thaw cycles were consistent with the overall trend in the freeze–thaw test with −17 °C freezing and 8 °C thawing, also the average diameter of bubbles increased, the bubble spacing factor increased and the bubble specific surface area decreased with the increase in the number of freeze–thaw cycles.

After 300 freeze–thaw cycles, the average diameter of bubbles in Group C concrete with 4% moisture content was 178 microns, the average diameter of bubbles in Group B concrete with 8% moisture content increased by 4.5% from Group C, and the average diameter of bubbles in Group A concrete with 15% moisture content increased by another 5.4% from Group B. The bubble spacing factor of Group C concrete was 206 µm, that of Group B concrete increased by 2.4% on the basis of Group C, and that of Group A concrete increased by 8.1% on the basis of Group B.

**Figure 10:**
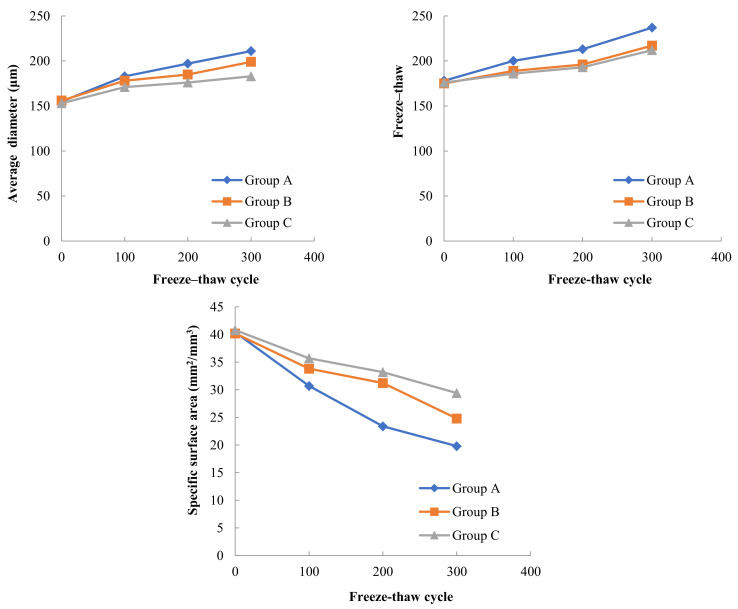
Variation of air void parameters for concrete with different internal water content (−40 °C freezing temperature).

The variation pattern of air void parameters with the number of freeze–thaw cycles for three Groups of concrete with different moisture contents in A, B and C under the freeze–thaw cycle test at −30 °C freezing low temperature and 8 °C melting temperature is shown in Figure 10.

Combining the results of the air void parameters tests in Figure 8, Figure 9 and Figure 10, it can be found that in addition to the internal humidity conditions of the concrete, the freezing temperature in the freeze–thaw cycle also has a large effect on the concrete air void parameters. At the same internal moisture content, the air void parameters in concrete will change with the decrease in freezing temperature. Taking Group A with 15% internal moisture content as an example, the average diameter of bubbles in concrete was 186 µm, 196 µm and 211 µm after 300 freeze–thaw cycles at three freezing low temperatures, including −17 °C, −30 °C and −40 °C, respectively. The lower the freezing temperature used in the freeze–thawing process, the larger the average diameter of the bubbles after freeze–thawing, and the effect of freezing temperature on the bubble spacing factor is similar to the average bubble diameter. The lower the freezing temperature, the smaller the specific surface area of the bubbles in the concrete after 300 freeze–thaw cycles.

Jie Yuan et al. [26] used more advanced tomographic CT combined with image processing to study the effect of freeze–thaw cycles on the air void parameters in hardened concrete under −17 °C freezing temperature and 8 °C thawing. Their results showed that the freeze–thaw action increased the diameter and spacing coefficient of air bubbles in concrete, and this phenomenon existed in both air-entrained and non-air-entrained concrete, and the variation rate of non-air-entrained concrete is several times that of air-entrained concrete. Their results are in general agreement with the findings of this paper at a freezing temperature of −17 °C, but they do not further explore the effect of reducing the freezing temperature during freeze–thaw cycles on the bubble parameters of hardened concrete. The principle that the introduction of air bubbles into concrete by air-entraining agents can improve the frost resistance of concrete has been well explained in the literature [27,28,29]. When an air-entraining agent is incorporated into concrete, a large number of uniformly distributed tiny bubbles are introduced, which generally have a relatively fixed average diameter, bubble spacing coefficient and specific surface area, due to the compressibility of the bubbles, the bubbles can relieve the expansion pressure generated by icing. At the same time, the bubbles can also accommodate the migration of free water when the pores in the concrete freeze, alleviating the infiltration pressure, these qualities of the bubbles play a key role in improving the frost resistance of concrete. When concrete with different internal water content is subjected to freeze–thaw cycles, concrete with high internal water content is more likely to form larger expansion pressures in concrete pores than concrete with low water content, and larger expansion pressures can cause rupture of tiny bubbles, which in turn can form bubbles with larger diameters. Along with the rupture of tiny bubbles to form larger diameter bubbles, the spacing factor of bubbles in concrete will also increase and the specific surface area of bubbles will decrease. Similarly, when concrete with the same internal water content is subjected to freeze–thaw cycles at different freezing temperatures, the lower freezing temperatures will also cause greater expansion pressure to form in the concrete pores, which in turn will increase the average diameter of the bubbles in the concrete, increase the bubble spacing factor, and decrease the specific surface area of the bubbles.

## 4. Conclusions

The frost resistance and air void parameters of concrete with different internal water content were studied under different freezing low temperature conditions during freeze–thaw cycles, and the main conclusions are as follows.

Before the freeze–thaw test, the average diameter of bubbles, bubble spacing factor and bubble specific surface area of concrete with different internal water content did not differ significantly. However, with the increase in the number of freeze–thaw cycles, the performance of concrete frost resistance and air void parameters gradually deteriorated, as shown by the increase in mass loss rate and the decrease in relative dynamic elastic modulus retention value, as well as the gradual increase in average bubble diameter and bubble spacing factor and the gradual decrease in bubble specific surface area. The deterioration of concrete air void parameters in the freeze–thaw process is mainly caused by the gradual decrease in tiny bubbles and the gradual increase in large bubbles. The test results show that the higher the internal water content of concrete, the more serious the performance deterioration of concrete air void parameters during freezing and thawing.

When the internal water content of concrete is the same, in the freeze–thaw cycle test, the freezing temperature decreases and the frost resistance of concrete becomes worse, and as the average diameter of bubbles and bubble spacing factor in concrete gradually increases, the specific surface area of bubbles gradually decreases. The lower the freezing temperature used in the freeze–thaw test process, the greater the expansion force in the concrete pores, which in turn leads to a gradual reduction in tiny bubbles and a gradual increase in large bubbles inside the concrete.

## Figures and Tables

**Table 1:** Concrete mix proportion.

Water Binder Ratio	Ordinary Portland Cement	Grade I Fly Ash	Natural Sand	Limestone Coarse Aggregate	Water-Reducing Agent (1/100)	Air-Entraining Agent (1/10,000)
0.38	1	0.25	2.45	4.55	0.9	5.0

**Table 2:** Conditions for further curing of concrete specimens.

Further Curing Condition			Sample Group
Temperature	Relative Humidity	Curing Time	
20 °C	90%	24 h	Group A
20 °C	60%	24 h	Group B
60 °C	60%	24 h	Group C

**Table 3:** Air void parameters test results.

Samples	Moisture Content (%)	Freeze Temperature (°C)	Average Diameter (μm)				Spacing Factor (μm)				Specific Surface Area (mm^2^/mm^3^)			
			0	100	200	300	0	100	200	300	0	100	200	300
Group A	15	−17	155	169	173	186	178	186	196	214	40.4	36.4	30.3	25.4
		−30		178	189	196		193	205	228		34.8	26.7	23.3
		−40		183	197	211		200	213	237		30.7	23.4	19.8
Group B	8	−17	156	162	168	175	175	180	186	204	40.2	38.5	35.4	30.2
		−30		171	179	186		184	190	211		36.7	33.5	27.5
		−40		178	185	199		189	196	217		33.8	31.2	24.8
Group C	4	−17	153	157	161	169	176	179	182	195	40.8	39.8	37.7	33.6
		−30		166	170	178		182	187	206		38.2	35.4	31.7
		−40		171	176	183		186	193	212		35.7	33.2	29.4
